# Exercise-Induced Fibroblast Growth Factor-21: A Systematic Review and Meta-Analysis

**DOI:** 10.3390/ijms24087284

**Published:** 2023-04-14

**Authors:** Hyunjoong Kim, Jihye Jung, Sungeon Park, Younglan Joo, Sangbong Lee, Jeongu Sim, Jinhyeong Choi, Hyun Lee, Gyujeong Hwang, Seungwon Lee

**Affiliations:** 1 Seogwangju Chung Yeon Rehabilitation Hospital, 61, Gaegeum-gil, Gwangju 72070, Republic of Korea; 2 Department of Physical Therapy, Gwangju Health University, 73, Bungmun-daero 419, Gwangju 62287, Republic of Korea; 3 Institute of SMART Rehabilitation, Sahmyook University, 815, Hwarang-ro, Seoul 01795, Republic of Korea; 4 Department of Physical Therapy, Graduate School, Sahmyook University, 815, Hwarang-ro, Seoul 01795, Republic of Korea; 5 Department of Physical Therapy, Sahmyook University, 815, Hwarang-ro, Seoul 01795, Republic of Korea

**Keywords:** fibroblast growth factor-21, exercise, endocrine cells, physical activity

## Abstract

This systematic review aimed to synthesize and quantify the results of the studies investigating the changes in fibroblast growth factor-21 (FGF-21) induced by exercise. We searched for studies that did not differentiate between patients and healthy adults but compared them before and after exercise and with and without exercise. For quality assessment, the risk-of-bias assessment tool for nonrandomized studies and the Cochrane risk-of-bias tool were used. A quantitative analysis was performed using the standardized mean difference (SMD) and random-effects model in RevMan 5.4. A total of 94 studies were searched in international electronic databases, and after screening, 10 studies with 376 participants were analyzed. Compared with no exercise, there was a significant increase in the FGF-21 levels from before to after exercise (SMD = 1.05, 95% confidence interval (CI), 0.21 to 1.89). The changes in FGF-21 levels in the exercise group showed a significant difference from the levels in the controls. The results of the random-effects model were as follows: SMD = 1.12; 95% CI, −0.13 to 2.37. While the data on acute exercise were not synthesized in this study, FGF-21 levels generally increased after chronic exercise compared with no exercise.

## 1. Introduction

Overweight status, obesity, metabolic syndrome, and diabetes due to lifestyle changes are the main risk factors for various chronic diseases [1,2]. The risk of developing obesity due to weight gain is predicted to be accelerated by 2–5 years [3]. These data indicate the importance of weight control, and it has been reported that exercise is an effective means of weight control [4].

The pancreatic hormone, glucagon, plays many roles in the body’s response to exercise [5], and glucagon receptor (GcgR) signaling is an important factor in exercise-induced metabolism [6]. With chronic exercise, GcgR activation induces the expression of fibroblast growth factor (FGF)-21, which has a positive effect on glucagon metabolism [7].

The FGF-21 has recently received increased attention, and it plays an important role in the regulation of secretory functions and metabolic processes [8]. It is a protein involved in glucose uptake, lipid metabolism, and energy balance [9]; is synthesized and secreted by the liver [10]; and is partially secreted by adipose tissue [11,12]. Reported effects include inhibition of gluconeogenesis, fatty acid oxidation, lipolysis, ketogenesis, lipogenesis, and blunting of the growth hormone signaling pathway [13,14,15]. In animal models, FGF-21 was reported to have an effect on glucose homeostasis [9,16,17,18] via improved insulin sensitivity, improved hypertriglyceridemia, and decreased fat production in the livers of obese diabetic rodents and rhesus monkeys. In human studies [19,20], FGF-21 contributes to plasma levels during exercise, and this, in turn, affects the glucagon:insulin ratio [21].

As research on human participants and exercise-related changes in FGF-21 levels has become more robust, this review identified studies on exercise-induced FGF-21 and conducted a systematic review with a meta-analysis, including qualitative and quantitative analyses.

## 2. Materials and Methods

### 2.1. Study Design

In this systematic review, we aimed to synthesize and qualitatively and quantitatively analyze studies conducted on exercise-induced changes in FGF-21 in human participants. This review was registered in the International prospective register of systematic reviews (PROSPERO, No. CRD42022328341), and it followed the preferred reporting items for systematic reviews and meta-analyses guidelines.

### 2.2. Search Strategy and Selection of Studies

#### 2.2.1. Inclusion Criteria

1. Participants:

Studies with human participants without consideration of medical conditions were included.

2. Intervention:

Interventions related to physical activity were included regardless of exercise type.

3. Comparisons:

Comparisons were not required in single-arm studies. Randomized controlled trials (RCTs) and non-RCTs included studies that showed the effects of physical activity and studies that did not include an intervention.

4. Outcomes:

Results included only studies measuring FGF-21.

5. Types of studies:

Both RCTs and non-RCTs that included physical activity and measured FGF-21 were included. In addition, in the case of a single group, before-and-after comparison studies were included.

#### 2.2.2. Exclusion Criteria

Studies for which data were not reported, studies that were not in English in the original text, and studies on animals were excluded.

#### 2.2.3. Strategy for Literature Search

By October 2022, the study protocol was registered in PROSPERO, and studies conducted before that time were included in this review. The following keywords were used to search various databases: (“fibroblast growth factor 21” OR “FGF-21”) AND (“exercise” OR “physical activity” OR “training”).

The following international electronic databases were searched: Excerpta Medica Database, Physiotherapy Evidence Database (PEDro), Cumulative Index to Nursing and Allied Health Literature, and Medical Literature Analysis and Retrieval System Online.

#### 2.2.4. Study Selection and Data Extraction

Among the studies searched through electronic databases, duplicate data were excluded, using a reference management software (EndNote 20, Thomson Reuters, New York, NY, USA). Researchers with experience in meta-analysis adopted the following procedure: first, check the title and abstract based on the inclusion criteria; second, check the study design and data of the original text; third, consult extracted and excluded studies; and fourth, extract data from the selected studies.

#### 2.2.5. Quality Assessment

The quality assessment process was different for different types of studies. For RCTs and controlled clinical trials, the risk of bias (RoB) was provided by RevMan 5.4 (The Cochrane Collaboration, Oxford, England) [22]. For other observational studies, assessment was performed using the risk-of-bias assessment tool for nonrandomized study (RoBANS) [23].

RoB classifies the risk of bias as low (−), high (+), and uncertain (?), and it consists of the following items: random sequence generation, allocation concealment, blinding of participants and personnel, blinding of outcome assessment, incomplete outcome data, selective reporting, and other biases. The RoBANS classifies the risk of bias as low, high, and uncertain and consists of the following items: selection of participants, confounding variables, measurement of exposure, blinding of outcome assessments, incomplete outcome data, and selective outcome reporting.

### 2.3. Strategy for Data Synthesis

The synthesis of the selected studies used RevMan 5.4 for quantification. Because changes in FGF-21 levels were analyzed, quantitative analysis was performed using standardized mean differences (SMD). In addition, considering the heterogeneity of different studies, we performed an analysis using a random-effects model. Chi-square and I^2^ tests were used to analyze the heterogeneity.

Interpretation of the I^2^ value, depending on the derived value, implied high heterogeneity if it was greater than 75% and low heterogeneity if it was less than 40% [24]. Publication bias was analyzed using a funnel plot [25].

## 3. Results

### 3.1. The Literature Search and Characteristics of the Included Trials

**Figure 1:**
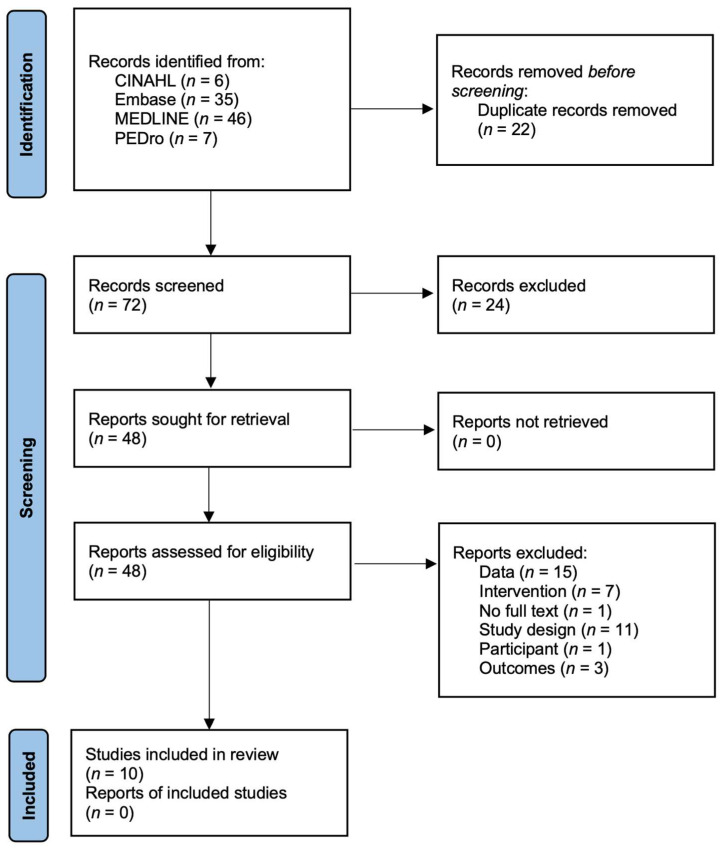
PRISMA flow diagram.

A total of 94 studies were identified using the search strategy. Twenty-two duplicate studies were identified by the reference management tool. Of the 72 studies, 24 were excluded after title and abstract review, and the original text of 48 studies was reviewed. After screening the studies according to the inclusion criteria, 38 studies were excluded, and finally, 10 studies were selected (Figure 1) [26,27,28,29,30,31,32,33,34,35].

### 3.2. Methodological Quality Assessment of Studies on Exercise-Induced FGF-21

**Figure 2:**
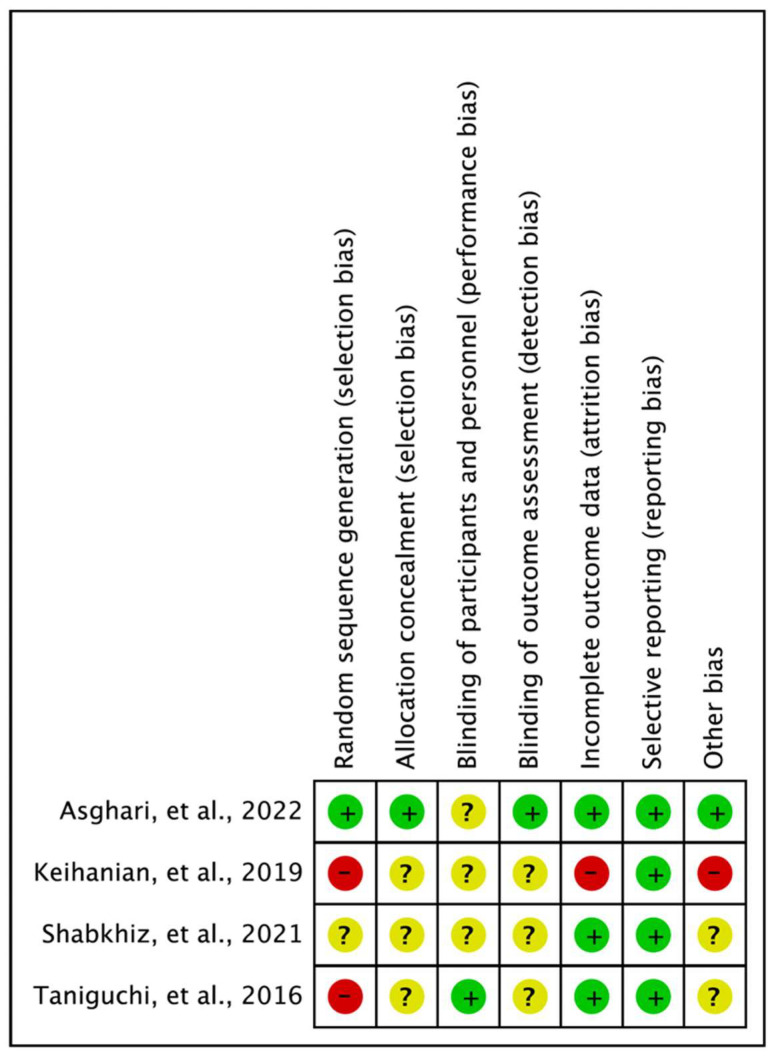
Risk of bias summary [29,30,33,35].

A pilot test was conducted for quality assessment, and the agreement rate was confirmed. The quality of four studies [29,30,33,35] was assessed using the RoB tool, and the results were as follows: random sequence generation (low, 1; uncertain, 2; high, 1), allocation concealment (low, 1; uncertain, 3), blinding of participants and personnel (low, 1; uncertain, 3), blinding outcome assessment (low, 1; uncertain, 3), incomplete outcome data (low, 3; high, 1), selective reporting (low, 4), and other biases (low, 1; uncertain, 2; high, 1) (Figure 2). Four studies [26,27,28,31,32,34] were assessed by the RoBANS, and the results were as follows: selection of participants (low, 4; uncertain, 1; high, 1), confounding variables (low, 1; uncertain, 4; high, 1), measurement of exposure (low, 5; uncertain, 1), blinding of outcome assessments (low, 4; uncertain, 2), incomplete outcome data (low, 5; uncertain, 1), and selective outcome reporting (low, 5; uncertain, 1) (Table 1).

### 3.3. Chronic Effects of Exercise on FGF-21

A total of 10 studies, involving 376 participants (patients and healthy adults), were analyzed in this review. The condition of the participants was not considered, and only studies in which the outcome was FGF-21 were included. Table 2 shows the contents of the 10 selected studies. Although consistent results were not obtained, resistance training decreased FGF-21 levels, while aerobic training increased FGF-21 levels. The duration of training was inconsistent; therefore, it was not possible to distinguish between the acute and chronic effects of exercise.

### 3.4. Within-Group Comparisons of Exercise-Induced FGF-21

**Figure 3:**
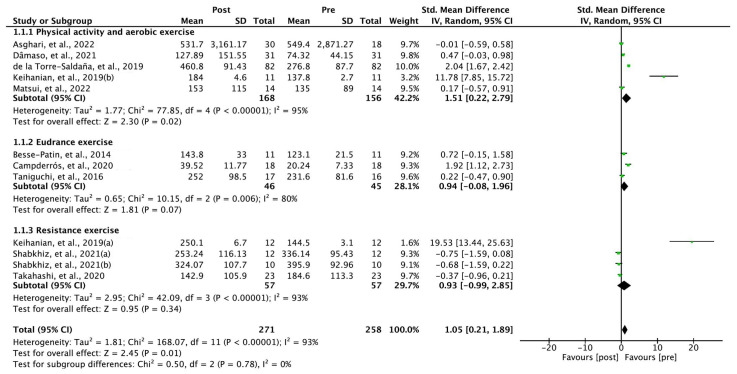
Forest plot of within-group comparisons of exercise-induced changes in fibroblast growth factor-21 levels [26,27,28,29,30,31,32,33,34,35]. Keihanian et al., 2019(a) [29]—resistance exercise; Keihanian et al., 2019(b) [29]—aerobic exercise; Shabkhiz et al., 2021(a) [30]—participants without type 2 diabetes; Shabkhiz et al., 2021(b) [30]—participants with type 2 diabetes.

The 10 studies included for comparison before and after exercise intervention within the group were quantitatively analyzed (Figure 3). The FGF-21 levels before and after exercise showed a significant difference. The results of the random-effect model were as follows: SMD = 1.05; 95% confidence interval (CI), 0.21 to 1.89; heterogeneity—χ^2^ = 168.07, df = 11, I^2^ = 93%; overall effect—Z = 2.45, *p* = 0.01. Upon analyzing the effects of both physical activity and aerobic exercise in the subgroup analysis, classified as intervention characteristics, the following results were obtained: SMD = 1.51; 95% CI, 0.22 to 2.79; heterogeneity—χ^2^ = 77.85, df = 4, I^2^ = 95%; overall effect—Z = 2.30, *p* = 0.02. Upon analyzing the effects of endurance exercise in the subgroup analysis, the following results were obtained: SMD = 0.94; 95% CI, −0.08 to 1.96; heterogeneity—χ^2^ = 10.15, df = 2, I^2^ = 80%; overall effect—Z = 1.81, *p* = 0.07. Upon analyzing the effects of resistance exercise in the subgroup analysis, the following results were obtained: SMD = 0.95; 95% CI, −0.99 to 2.85; heterogeneity—χ^2^ = 42.09, df = 3, I^2^ = 93%; overall effect—Z = 0.95, *p* = 0.34.

### 3.5. Exercise-Induced FGF-21 Levels in Comparison with Controls

**Figure 4:**
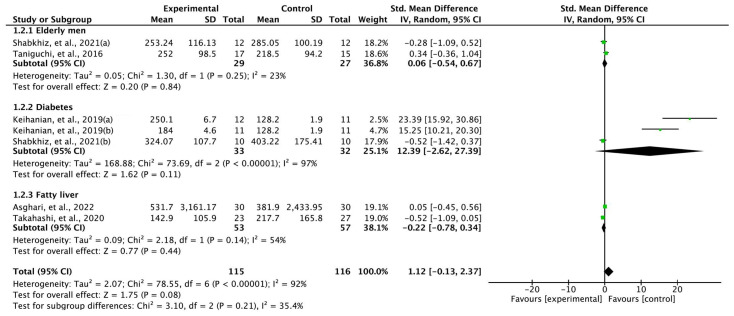
Forest plot of between-group comparisons, i.e., intervention and control groups, of changes in exercise-induced fibroblast growth factor-21 levels [29,30,31,33,35]. Keihanian, et al., 2019(a) [29]—resistance exercise; Keihanian et al., 2019(b) [29]—aerobic exercise; Shabkhiz, et al., 2021(a) [30]—without type 2 diabetes; Shabkhiz et al., 2021(b) [30]—with type 2 diabetes.

Four studies were quantitatively analyzed for the comparison of exercise and control groups (Figure 4). The changes in FGF-21 levels in the exercise group showed a significant difference from the levels in the controls. The results of the random-effects model were as follows: SMD = 1.12; 95% CI, -0.13 to 2.37; heterogeneity—χ^2^ = 78.55, df = 6, I^2^ = 92%; and overall effect—Z = 1.75 (*p* = 0.08). Furthermore, a subgroup analysis was performed based on the health condition of the participants. Elderly men: SMD = 0.06; 95% CI, −0.54 to 0.67; heterogeneity—χ^2^ = 1.30, df = 1, I^2^ = 23%; overall effect—Z = 0.20 (*p* = 0.84); diabetes—SMD = 12.39; 95% CI, −2.62 to 27.39; heterogeneity—χ^2^ = 73.69, df = 2, I^2^ = 97%; overall effect—Z = 1.62 (*p* = 0.11); fatty liver—SMD = −0.22; 95% CI, -0.78 to 0.34; heterogeneity—χ^2^ = 2.18, df = 1, I^2^ = 54%; overall effect—Z = 0.77 (*p* = 0.44).

### 3.6. Publication Bias

Ten studies were included. No publication bias was reported because fewer than 10 studies were included according to the recommendations of the Cochrane Review [36].

## 4. Discussion

To our knowledge, this is the first systematic review and meta-analysis to quantify chronic-exercise-induced FGF-21. Exerkines are humoral factors secreted from each organ into circulation in response to acute or chronic exercise [37]. Among these, FGF-21 is an exerkine [38] that plays an important role in glucose homeostasis and metabolic regulation. Therefore, we attempted to determine how much FGF-21 is induced by exercise and what kind of exercise induces the said changes.

This review included four RCTs and six single-arm intervention studies. The results showed that a significant change in FGF-21 levels occurred before and after exercise (SMD = 1.05; 95% CI, 0.21 to 1.89). As a result of analyzing the changes according to the type of exercise through subgroup analysis, aerobic exercise, physical activity, and endurance exercise induce greater changes than resistance exercise. A meta-analysis of RCTs showed a significant change in FGF-21 levels (SMD = 1.12; 95% CI, −0.13 to 2.37). In addition, in the subgroup analysis, classification was possible according to health conditions, and as a result, more changes were induced in diabetes than in the elderly or fatty liver.

Our synthesized results confirmed that exercise alone could induce changes in FGF-21 levels, and there was a difference in the extent of changes depending on the exercise type. In the case of resistance exercise, FGF-21 levels decreased after exercise in all but one study [29]. In a study on older adults, resistance exercise was reported to be effective in ensuring glycemic control and insulin resistance [39,40]. This result is contrary to our results, and there is a limit to the generalization of the cases studied herein. Not only are the molecular mechanisms not fully understood, but they are also only partially explained by the results of exercise. In addition, both physical activity and aerobic exercise significantly increased the FGF-21 levels. As mentioned in the Introduction section, aerobic exercise and FGF-21 can improve glucose homeostasis, regulate lipid utilization, increase thermogenesis in brown fat, and improve insulin resistance; they are also associated with browning of the white adipose tissue [41,42,43]. This is similar to endurance exercise in the characteristics of exercise.

Therefore, based on the subgroup analysis results, the changes in FGF-21 compared with recent studies are as follows. By type of exercise: aerobic exercise; increase, endurance exercise; increase, resistance exercise; decrease. By health condition: elderly; conflict, diabetes; increased, fatty liver; conflict. Based on previous studies, FGF-21 mainly showed an increase in aerobic exercise and endurance exercise, which is consistent with a clearer result as the age is younger and thinner [44,45]. It was decreased in resistance exercise, but other previous studies showed conflicting results. Conflicting results of increasing or decreasing the effect of chronic exercise are considered to be a decrease in FGF-21 by adaptation, such as reducing insulin resistance [45]. Moreover, it can be attributed to the change in intensity, not the type of exercise [46,47]. Regarding the difference according to health conditions, the elderly showed conflicting results. This is consistent with the fact that it increases with age [45]. Results in diabetes and fatty liver are predictable when considering glycolipid homeostasis and deterioration of liver function.

For the abovementioned results, the mechanism underlying the changes in the FGF-21 level was explained in relation to effect of chronic exercise but not acute exercise. In a systematic review on acute exercise, it was reported that FGF-21 increased regardless of body weight; the increase was maintained for 1 h and returned to a value close to the baseline after 3 h [48]. However, it has been reported that chronic exercise induces adrenaline activation and, thus, leads to an increase in FGF-21 levels, while acute exercise cannot cause such changes [49]. However, the effects of acute exercise were not synthesized, owing to differences in the literature search strategy. The significant changes in FGF-21 levels caused by chronic exercise were observed in our review mainly because exercise produces beneficial effects through an increase in physical activity via the consumption of lipids and carbohydrates. In addition, lifestyle-related diseases [50], such as metabolic syndrome, diabetes, and obesity, have been reported to be caused by decreased physical activity levels. A previous study reported a direct relationship between physical activity and FGF-21 levels (*r* = 0.23, *p* = 0.002) [51]. In addition, long-term and regular physical activity over 6 months can lead to changes at the cellular level through lifestyle changes [52].

The type of exercise and effects of chronic exercise on FGF-21 levels were partially explained through the synthesized results, and the mechanisms have been elucidated. Essentially, the mechanisms underlying the relationship between exercise and FGF-21 are as follows: Exercise-induced FGF-21 activates insulin [53] and contributes to enhanced muscle glucose metabolism upstream of AMP-activated kinase activity [16]. This process occurs because FGF-21 stimulates mitochondrial biosynthesis and increases the oxidative capacity of myofibrils [54,55]. In addition, an increase in FGF-21 levels in lipid homeostasis induces a decrease in the level of free fatty acids [56], thus preventing the ectopic deposition of lipids in the liver and muscles. Consequently, an increase in FGF-21 levels leads to improved systemic glucose and lipid homeostasis [57].

Additionally, FGF-21 has been reported to affect brain health. First, in previous studies, brain-derived neurotrophic factor (BDNF) was the most frequently reported exerkine. BDNF is a protein that plays an important role in neuronal development, plasticity, differentiation, and survival [58]. A meta-analysis reporting changes in BDNF through aerobic exercise showed improvements in BDNF levels in older adults with mild cognitive impairment (SMD = 0.48) [59]. Interestingly, in addition to BDNF, FGF-21 also has an impact on brain health [60]. Although evidence on the mediation effect of FGF-21 on the induction of brain plasticity is unclear, its direct neuroprotective effects on neurons have been reported [60,61]. In addition, a potential central action of FGF-21 has been reported, namely that peripheral FGF-21 can cross the blood–brain barrier in mice; it has been detected in human cerebrospinal fluid [62,63]. However, few studies have been conducted so far, so only hypotheses have been reported, and further investigations are needed.

Furthermore, in relatively recent studies, interesting studies related to the cardiovascular system have been published. FGF-21 induced the AMPK/FOXO3/SIRT3 signaling axis in stem-cell-derived cardiomyocytes. This resulted in preventing mitochondrial dysfunction and oxidative stress [64]. From a new point of view, biological functions were also found to be performed in the vascular unit, and it was reported to reduce the risk of bleeding by inhibiting thrombus formation [65].

This systematic review had several limitations, and future study directions are as follows: First, although the synthesized results were interesting, the heterogeneity was too high. Second, we performed a subgroup analysis of patients and healthy adults, so the results had limited reliability. Third, physical activity refers to movement of the body in which skeletal muscles are used, but it also includes lifestyle interventions; hence, a diet program was included, which may be a factor that increases heterogeneity. Fourth, although the effect of acute exercise was not synthesized, the effect of acute exercise according to the condition of the participants should be investigated. Fifth, the participants were classified according to the exercise type, and time spent training before study participation was not consistent. Lastly, it was worth investigating the effect of FGF-21 not only on glucose homeostasis but also the other diseases group, such as those associated with brain plasticity.

## 5. Conclusions

This study looked at the chronic effects of exercise-induced changes in FGF-21 levels. Aerobic exercise, physical activity, and endurance exercise could be selected as effective exercises for changes in FGF-21 levels, and depending on health conditions, it could be more induced in diabetic patients than in the elderly or fatty liver.

## Figures and Tables

**Table 1:** Risk-of-bias assessment tool for non-randomized studies.

Items	Besse-Patin et al., 2014 [34]	Campderrós et al., 2020 [26]	Dâmaso et al., 2021 [27]	De la Torre-Saldaña et al., 2019 [28]	Matsui et al., 2022 [32]	Takahashi et al., 2020 [31]
Selection of participants	Low	Low	Low	Unclear	Low	High
Confounding variables	Low	Unclear	Unclear	Unclear	Unclear	High
Measurement of exposure	Low	Low	Low	Low	Low	Unclear
Blinding of outcome assessments	Unclear	Low	Low	Low	Unclear	Low
Incomplete outcome data	Low	Low	Low	Low	Unclear	Low
Selective outcome reporting	Low	Low	Low	Low	Unclear	Low

**Table 2:** Characteristics of included studies.

Study	Health Condition (*n*) Participants (Mean Age)	Study Design EG and/or CG	Therapeutic Intensity	Conclusion
Asghari et al., 2022 [33]	NAFLD (*n* = 60) CR (40.08 years) and CG (39.27 years)	A randomized controlled trial CR: healthy calorie-restricted diet CG: control	Healthy eating and weight control advice for12 weeks Participants in the CR group were targeted to lose a maximum of 10% of their baseline body weight through a healthy calorie-restricted diet	CR diet with moderate weight loss has some favorable effects on NAFLD but was not able to modify oxidative/antioxidative status in these patients.
Besse-Patin et al., 2014 [34]	Eleven obese (*n* = 11) Non-diabetic male subjects (35.4 years)	An interventional clinical trial Single arm: endurance training	8-week endurance training The 45–60 min exercise sessions consisted mainly of cycling and running, 5 times a week, for 8 weeks	Exercise training upregulates muscle apelin expression in obese subjects.
Campderrós et al., 2020 [26]	Healthy (*n* = 18) Marathon runners (41.71 years)	An interventional clinical trial Single arm: marathon	42.2-km running race Maintaining an adequate level of hydration during the race	GDF-15 and FGF-21 levels transiently increased in runners following a marathon race.
Dâmaso et al., 2021 [27]	Overweight and obese (*n* = 31) Overweight and obese women (32 years)	An interventional clinical trial Single arm: overweight and obese women	12-week interdisciplinary weight loss program Nutritional therapy (individual nutritional consultation), physical activity (weekly videos with examples of exercise and health education information), and education for lifestyle changes	Changes in FGF-21 concentrations were different among the women participating in the weight loss program, with some having increased levels and some reduced levels.
De la Torre-Saldaña et al., 2019 [28]	Healthy (*n* = 82) Young sedentary healthy women (23 years)	An interventional clinical trial Single arm: physical activity	Maintaining daily physical activity according to a regular diet, lifestyle, and instructions for 2 weeks	Serum irisin and FGF-21 levels significantly increased after 2 weeks of supervised physical activity.
Keihanian et al., 2019 [29]	Type 2 diabetes mellitus (*n* = 34) ATG (52.4 years), RTG (52.4 years), and CG (53.0 years)	A controlled clinical trial ATG: aerobic training RTG: resistance training CG: control	Aerobic training: 30–45 min of aerobic running at 65–75% of maximum heart rate for 8 weeks Resistance training: 8 weeks of three sets of 10 repetitions maximum of leg press, bench press, knee extension, seated cable row, knee flexion, military press, and calf rise.	Aerobic and resistance exercise training led to a significant decrease in serum fetuin-A and fetuin-B levels and increased FGF-21 levels in men with type 2 diabetes mellitus.
Matsui, et al., 2022 [32]	Overweight and obese (*n* = 14) Overweight and obese men (49 years)	An interventional clinical trial Single arm: aerobic exercise	Supervised aerobic exercise training for 12 weeks (three times per week) Aerobic exercise (walking and/or jogging) was performed with moderate intensity (Borg scale: 12–14) for approximately 40–60 min.	Lowering postprandial circulating FGF21 levels may be associated with the improved glucose tolerance induced by habitual aerobic exercise in overweight and obese men.
Shabkhiz et al., 2021 [30]	Elderly men with and without T2D (*n* = 44) Elderly men without T2D (72.08 years) and with T2D (72.45 years)	A randomized controlled clinical trial EG: resistance training without and with T2D CG: normal activity without and with T2D	Resistance training: machine-based exercises (leg press, leg extension, seated leg curl, seated calf, bench press, compound row, triceps press, and bicep curl) over 12 weeks/3 sessions per week.	12 weeks of RT induced an overall significant reduction of FGF-21 and myostatin in elderly men with and without T2D.
Takahashi et al., 2020 [31]	NAFLD (*n* = 50) EG (55.5 years) and CG (50.4 years)	A retrospective clinical study EG: resistance training CG: lifestyle counseling	Resistance training: three sets of push-ups and three sets of squats at 20–30 min per session 3 times a week for a total of 12 weeks.	Simple resistance exercise reduced CK-18 and FGF-21 levels in patients with NAFLD.
Taniguchi et al., 2016 [35]	Elderly men (*n* = 32) Elderly Japanese men (69.6 years)	A randomized crossover trial EG: endurance exercise CG: control	5-week endurance exercise program The exercise program comprised three cycle ergometer sessions per week. The exercise time was 30 min for weeks 1 and 2 and 45 min for weeks 3–5.	A 5-week endurance exercise program decreased hepatic fat content and serum FGF21 levels without weight loss in elderly men, and exercise-induced hepatic fat reduction mediated the reduction in serum FGF21 levels.

ATG, aerobic training group; CG, control group; CK-18, cytokeratin 18; CR, calorie-restricted; EG, experimental group; FGF-21, fibroblast growth factor 21; GDF-15, growth differentiation factor 15; NAFLD, nonalcoholic fatty liver disease; RTG, resistance training group; T2D, type 2 diabetes mellitus.

## Data Availability

Not applicable.
